# Assessment of Tear Osmolarity in Smokers Using TearLab and I-Pen Systems

**DOI:** 10.1155/2022/9970388

**Published:** 2022-10-27

**Authors:** Mana A. Alanazi, Gamal A. El-Hiti, Osamah A. Alturki, Muteb Alanazi, Raied Fagehi, Ali M. Masmali

**Affiliations:** Cornea Research Chair, Department of Optometry, College of Applied Medical Sciences, King Saud University, Riyadh 11433, Saudi Arabia

## Abstract

**Purpose:**

Smoking has a negative effect on health and ocular tear film. The purpose of the current study is to investigate the correlation between the TearLab and I-Pen osmolarity scores in smokers and compare them with those of non-smoking healthy males.

**Methods:**

Thirty male smokers (25.3 ± 2.2 years) participated in the study. An age-matched (22.9 ± 2.0 years) control group of non-smoking healthy males (*N* = 30) was also recruited for comparison purposes. The ocular surface disease index (OSDI) was completed first, followed by the TearLab and I-Pen osmolarity measurements.

**Results:**

The median TearLab osmolarity score was lower (*P* < 0.001) than that obtained using the I-Pen in both the study and the control groups. The OSDI scores and osmolality measurements were higher (*P* < 0.001) in smokers than in the control subjects. In the smoker group, there were moderate correlations between the OSDI scores and the measurements obtained using the TearLab (Spearman's correlation coefficient, *r* = 0.463; *P* = 0.010) and I-Pen (*r* = 0.449; *P* = 0.013) systems. In addition, there was a strong correlation between the osmolarity scores obtained from the TearLab and I-Pen systems in smokers (*r* = 0.911; *P* < 0.001).

**Conclusion:**

The I-Pen scores in smokers were significantly higher than those obtained using TearLab. The TearLab scores showed small variations compared with those obtained using I-Pen. A strong correlation was found between the TearLab and I-Pen scores in smokers. The osmolarity TearLab and I-Pen scores were significantly higher in smokers compared with normal eye subjects.

## 1. Introduction

Cigarettes contain many toxic components, such as heavy metals (e.g., cadmium, arsenic, chromium, lead, and nickel) [1]. In addition, burning cigarettes leads to the production of many toxic materials, such as nicotine, carbon monoxide, tar, volatiles, heavy hydrocarbons, amines, acids, and aldehydes [2]. The accumulation of toxicants inside the human body leads to serious damage and illnesses. These toxicants cause cancer [3], damage the renal and cardiovascular systems [4], and negatively affect the central nervous system [5]. Smoking shortens the human lifespan by at least 10 years and increases the risk of death threefold compared with non-smokers [6, 7]. Deaths due to the consumption of tobacco amount to more than 7 million worldwide per year [8]. Annually, smoking costs more than $300 billion, including health care and productivity loss as a result of premature death [6]. Therefore, regulations for the consumption of tobacco products have recently become stricter [6–8].

Smoking has a negative effect on the ocular tear film [9–12] as it leads to damage to the corneal epithelial layer and ocular surface [13]. It is also responsible for various ocular disorders, such as cataracts, glaucoma, thyroid eye, conjunctival intraepithelial neoplasia, and dry eye [14]. In addition, smoking is associated with dyslipidaemia and increases blood cholesterol levels, which are risk factors for dry eye [15, 16].

Various reports have established an association between smoking and dry eye [17–22]. Dry eye symptoms include irritation, inflammation, grittiness, scratchiness, foreign body sensation, and light sensitivity [23]. Dry eye is the result of a high evaporation rate and/or low tear volume [24]. Dysfunction of the meibomian gland is one of the most common causes of dry eye and is a result of lipid secretion shortage [25]. The methods used to detect dry eye include Schirmer's test [26], the phenol red thread (PRT) test [26], tear break-up time (TBUT) [27], tear evaporation rate [28], tear ferning [29–31], and the tear osmolarity [32] test, in addition to questionnaires, such as the ocular surface disease index (OSDI) [33].

Excessive tear evaporation leads to hyperosmolarity. Osmolarity measurement *in vivo* is a reliable tool to diagnose dry eye, especially since the introduction of the TearLab and I-Pen osmometers [34–38]. These osmometers do not induce reflux tears and require a very small volume of tears. In addition, the measurement procedure is simple, fast, repeatable, and accurate.

The *in vivo* correlation between osmolarity scores from the TearLab and I-Pen osmometers in normal eye subjects has been investigated [39]. The current prospective, non-randomized comparative study investigates the correlation between the TearLab and I-Pen osmolarity scores in smokers and compares them with those of non-smoking healthy males.

## 2. Subjects and Methods

Thirty male smokers ranging in age from 22 to 30 years (mean ± standard division (SD) = 25.3 ± 2.2 years) participated in the study. An age-matched (20–28 years; 22.9 ± 2.0 years) control group of non-smoking healthy males (*N* = 30) was also recruited for comparison purposes. Contact lens wearers and subjects with thyroid gland disorders, high blood cholesterol (above 4 mmol/L), high body mass index (above 24.9 kg/m^2^), refractive errors, vitamin A and D deficiencies, hypertension, anaemia, diabetes, or a history of ocular surgery were excluded from the study. This was an observational, case-control, and non-randomized comparative study. Ethical approval was obtained from the Ethics Committee of King Saud University (E-22-6803). The subjects were treated based on the Declaration of Helsinki. The participants signed written informed consent forms before the study.

The OSDI was completed first, followed by the use of the TearLab and I-Pen systems. The osmolarity measurement was carried out once on the right eyes by the same examiner. The test was performed only one time since the test has been proven to be repeatable and reproducible [32]. In addition, no variation was detected when the test was performed on the left eye. The TearLab system was used to measure osmolarity before the I-Pen system and the order was kept for all subjects. A gap of 5 minutes was allowed between the osmolarity measurements [32]. The measurements were performed in an air-conditioned clinic in which the temperature was set at 22°C and the humidity was less than 15% to minimize the variations and inaccuracy in the osmolarity readings. The tests were carried out in the morning session at the college clinics.

The OSDI (English version) was completed by all participants. The cutoff score for dry eye was >13 [33]. The TearLab osmolarity system was obtained from TearLab Corporation (San Diego, CA, USA). To ensure system functionality, electronic check cards were used to test the system (334 ± 4 mOsm/L) daily prior to its use [32]. The system uses a small sample of tears (50 nL) collected from the lateral lower tear meniscus. The countertop unit analyses the tear sample and displays the osmolarity score on a digital screen. The I-Pen osmolarity system was obtained from I-MED Pharma Inc. (Dollard-des-Ormeaux, Quebec, Canada). The system was used at a distance from any electronic devices to reduce the inaccuracy of the readings. Subjects were asked to close their eyes for 30 seconds, and the tip of the strip (disposable sensor) was held at a 30-degree angle in contact with the lower eyelid of the palpebral conjunctiva. A few seconds later, after a beep was heard, an osmolarity reading was displayed on the digital screen [36, 40]. The cutoff osmolarity score for the TearLab and I-Pen systems was 308 mOsm/L [41].

Microsoft Excel 2016 (Microsoft Corporation; Redmond, WA, USA) was used to collect data. Data were analysed using the Statistical Package for the Social Sciences software (version 22, IBM Software; Armonk, NY, USA). Spearman's correlation coefficient (*r*) was used to describe the correlation strength between different parameters [42]. The OSDI and osmolarity scores were not normally distributed (Kolmogorov–Smirnov test; *P* < 0.05); therefore, the Mann–Whitney *U* test (*P* < 0.05) was used to analyse the data. The median (interquartile range; IQR) was used to represent the average score for the OSDI and osmolarity measurements. The Bland–Altman analysis was used to test the agreement between the osmolarity measurements using TearLab and I-Pen systems in the smoker and non-smoker groups [43].

## 3. Results

The median (IQR) OSDI scores and osmolarity measurements for subjects in the study and control groups are reported in Table 1. In the study group, the OSDI scores ranged from 8.3 to 13.6 and indicated dry eye symptoms in only one subject. For the control group, the OSDI scores ranged from 0 to 10.4 with no symptoms of dry eye recorded. The median TearLab score was significantly (*P* < 0.001) lower than that obtained using I-Pen in both the study and the control groups. The OSDI scores and osmolality measurements were significantly higher (*P* < 0.001) in smokers than in the control group. For smokers, the TearLab and I-Pen scores ranged from 295 to 320 mOsm/L and from 302 to 337 mOsm/L, respectively. For the control group, the TearLab and I-Pen scores ranged from 263 to 304 mOsm/L and from 278 to 317 mOsm/L, respectively.

For smokers, the TearLab scores showed dry eye symptoms in 19 subjects (63.3%), while the I-Pen scores showed dry eye symptoms in 25 subjects (83.3%). For the control group, the I-Pen scores revealed that 16.7% of the subjects (*N* = 5) had dry eye symptoms. The TearLab scores indicated none of the subjects in the control groups had dry eyes.

**Figure 1:**
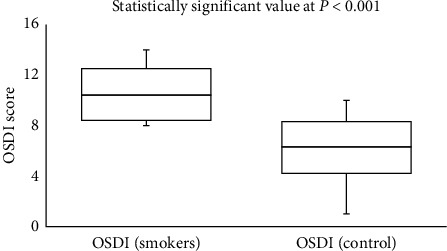
Side-by-side boxplot for OSDI scores for subjects in the study (smokers) and control (non-smokers) groups.

**Figure 2:**
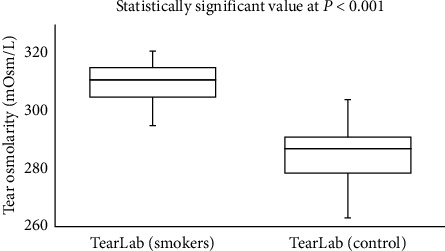
Side-by-side boxplot for tear osmolarity measurements using the TearLab system in the study (smokers) and control (non-smokers) groups.

**Figure 3:**
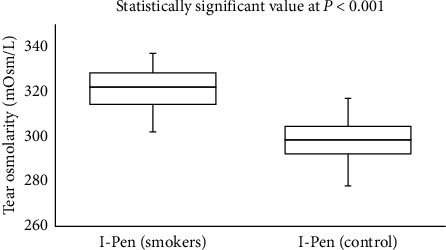
Side-by-side boxplot for tear osmolarity measurements using the I-Pen system in the study (smokers) and control (non-smokers) groups.

**Figure 4:**
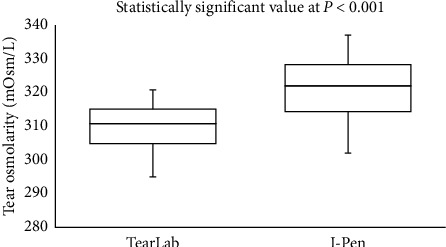
Side-by-side boxplot for tear osmolarity measurements using the TearLab and I-Pen systems in the study (smokers) group.

In the smoker group, there was a moderate correlation (Spearman's correlation coefficient, *r*) between the scores obtained from the OSDI scores and from TearLab (*r* = 0.463; *P* = 0.010) and I-Pen (*r* = 0.449; *P* = 0.013). Side-by-side boxplots for the OSDI scores for the study and control groups are shown in Figure 1. Figures 2 and 3 show the side-by-side boxplots for the TearLab and I-Pen scores in the study and control groups, respectively.  Figure 4 shows a side-by-side boxplot for the TearLab and I-Pen scores in smokers.

**Figure 5:**
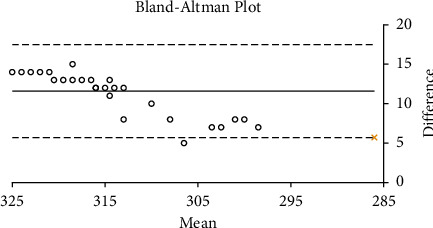
Bland–Altman plot for the correlation between tear osmolarity measurements taken using the TearLab and I-Pen systems in the study (smokers) group.

**Figure 6:**
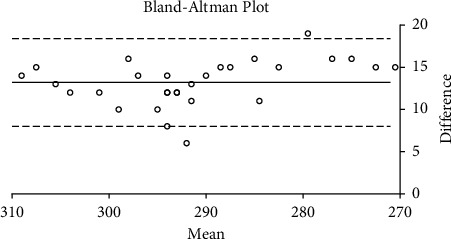
Bland–Altman plot for the correlation between tear osmolarity measurements taken using the TearLab and I-Pen systems in the control (non-smokers) group.

The Bland–Altman plots for the correlation between the TearLab and I-Pen osmolarity scores in the smokers and non-smokers are shown in Figures 5 and 6, respectively. There was a strong correlation between the TearLab and I-Pen osmolarity scores in smokers (*r* = 0.911; *P* < 0.001). On the other hand, there was a weak correction (*r* = 0.358; *P* < 0.05) between the measurements in the control group. However, a strong correlation was found between the osmolarity measurement scores using TearLab and I-Pen systems in both the smoker (*r* = 0.963; *P* < 0.001) and non-smoker (*r* = 0.972; *P* < 0.001) groups, based on Spearman's correlation coefficient.

## 4. Discussion

Smoking has a significant negative effect on the ocular tear film. Smoking reduces tear film stability and increases corneal staining [9]. In addition, eye irritation and dryness are common symptoms in smokers and passive smokers [13, 14, 17, 18]. Tear osmolarity scores can be used to detect the signs and severity of dry eye [44]. High tear osmolality is an indication of dry eye symptoms [45].

In this study, osmolarity scores were significantly higher in smokers compared to individuals in the control group. In smokers, significant changes occur within the lipid layer, possibly due to a peroxidation process [12, 14, 17]. These changes lead to an abnormal lipid spread [17]. As a result, the TBUT has been shown to be lower (5.4 s; *P* < 0.05) in smokers compared to control subjects (11.2 s) [17]. In addition, smoking leads to a high concentration of carbon monoxide in serum haemoglobin [14]. The level of carbon monoxide has been found to be 4.8% ± 0.4% in smokers and 0.5% ± 0.5% in control subjects [14]. Goblet cells have been found to be lower [14] and the tear evaporation rate has been found to be higher in smokers, compared with normal eye subjects [14, 28]. Moreover, smoking affects tear protein patterns, leading to tear film instability [46]. As such, the association between dry eye and smoking has been clearly established. The median OSDI score in the current study was comparable to those reported for smokers and subjects with a high body mass index [18, 47], but lower compared with the OSDI scores for subjects with diabetes and refractive errors [48–50].

The current study confirmed the presence of a strong correlation between the osmolarity measurements taken using the TearLab and I-Pen systems in smokers. However, the Bland–Altman plot shows a weak correction between the measurements in non-smokers. The reason for this observation could be due to the difference in the distribution of the osmolarity scores in the study and control groups. The median osmolarity scores using the TearLab and I-Pen systems were higher (*P* < 0.001) in smokers compared to non-smokers. As such, we suggest an association between dry eye symptoms (high osmolarity and ODSI scores). Additionally, osmolarity measurements taken using I-Pen were higher (*P* < 0.001) than those recorded using TearLab; these results are consistent with previous reports [39, 41]. Tear osmolarity measurements taken using TearLab have been shown to have better accuracy than those taken using the I-Pen system [41]. Previous research has demonstrated that the percentage of the coefficient of variation (CV%) for tear osmolarity measurements varies from 1.2% to 2.4% for the TearLab system, whereas the CV% is much higher (6.1%–6.4%) for tear osmolarity measurements taken using the I-Pen system [41]. Another study, conducted on 20 subjects with normal eyes, showed that the average tear osmolarity measured using I-Pen (319.4 ± 20.3 mOsm/L) was higher (*P* < 0.001) than that measured using the TearLab system (295.4 ± 8.6 mOsm/L) [39].

The variation in osmolarity scores measured using the two systems may be due to the high sensitivity of both systems, the I-Pen in particular, to several parameters, such as temperature and nearby motion [51–55]. Indeed, impedance measurements have been shown to affect tear temperature, leading to variation in osmolarity scores [54, 55]. A review of tear osmolarity measurements using the TearLab system showed high score variability among normal eye subjects [56]. However, tear osmolality measurements (299.1 ± 7.7 mOsm/L) using the TearLab system showed no significant differences among three readings from the same eye in healthy subjects (*N* = 30; 17 females and 13 males); the CV of this cohort ranged from 0.2% to 2.8%, with an average CV of 0.8% [18]. Additionally, the osmolarity measurements of traceable solutions at different temperatures using the I-Pen system have been shown to range from 286.6 to 298.2 mOsm/L, with a CV of 0.8% [36]. It has been suggested that the temperature coefficient factor is 2 mOsm/L per degree Celsius [36].

A previous study of 30 smokers suggested an association between smoking and dry eye symptoms [18]. For example, scores from the McMonnies questionnaire and tear ferning tests were significantly higher in smokers (9.83 ± 5.22 and 0.96 ± 0.54, respectively) compared with control subjects (5.96 ± 3.06 and 0.42 ± 0.38, respectively) [18]. Another study of 50 smokers who had smoked cigarettes for at least 5 years showed that tear osmolarity scores taken using the TearLab system were higher in smokers (305 ± 9.8 mOsm/L; *P* = 0.014) than in control subjects (301.1 ± 7.0 mOsm/L) [57]. In addition, the TBUT and goblet cell density were lower in smokers (8.1 ± 3.5 s and 18.8 ± 15.5 cells/mm^2^, respectively; *P* < 0.001) than in control subjects (13.7 ± 4.7 s and 31.2 ± 25.7 cells/mm^2^, respectively) [18]. However, no significant difference was found in tear volume, as measured using Schirmer's test, in smokers and control subjects [57]. In a number of studies, scores obtained from Schirmer's test and the PRT test were not conclusive, and the effect of smoking on tear volume is not clear [13, 17, 18, 58]. Each eye test assesses a specific parameter, and the correlation between different tests is generally poor [59].

The study has some limitations. No females were included in the study, the participants were from Riyadh City, and the study did not cover other areas in Saudi Arabia.

## 5. Conclusions

The I-Pen scores in smokers were significantly higher than those obtained using TearLab. The TearLab scores showed small variations compared with those obtained using I-Pen. A strong correlation was found between the TearLab and I-Pen scores in smokers. The osmolarity TearLab and I-Pen scores were significantly higher in smokers compared with normal eye subjects.

## Figures and Tables

**Table 1:** Median (IQR) OSDI and osmolarity scores for subjects in the study and control groups.

Parameter	Study group (*N* = 30)	Control group (*N* = 30)	*P* value^*∗*^
OSDI	10.4 (2.0)	6.3 (4.0)	<0.001
TearLab (mOsm/L)	310 (9.5)	287.0 (12.5)	<0.001
I-Pen (mOsm/L)	322.0 (14.0)	298.5 (12.3)	<0.001

^∗^Significant difference (Mann–Whitney test; *P* < 0.001).

## Data Availability

The data used to support the findings of this study are included within the article.
